# Glycemic Control and Quality of Life Among People with Type 1 Diabetes: Relationships with Insulin Therapy and Carbohydrate Counting

**DOI:** 10.3390/nu17121951

**Published:** 2025-06-06

**Authors:** Rui Jorge Dias, João Sérgio Neves, Rui Poínhos

**Affiliations:** 1 Faculty of Nutrition and Food Sciences, University of Porto, 4150-180 Porto, Portugal; ruijorgediasnutricionista@hotmail.com; 2 Faculty of Medicine, University of Porto, 4200-319 Porto, Portugal; jsneves@med.up.pt; 3 Local Health Unit of São João, 4200-319 Porto, Portugal; 4 Department of Biology and Environment, School of Life and Environmental Sciences, University of Trás-os-Montes e Alto Douro, 5000-801 Vila Real, Portugal

**Keywords:** glycemic control, quality of life, glycosylated hemoglobin, Diabetes Health Profile, eating self-efficacy

## Abstract

**Background/Objectives**: Diabetes mellitus has physical, psychological and behavioral implications related to glycemic control and quality of life. Our aims were to evaluate glycemic control and disease-related quality of life among adults with type 1 diabetes mellitus and to study their relationships with sociodemographic, clinical and anthropometric characteristics. **Methods**: Eighty-five participants (49.4% females, mean age 36 years, mean BMI 25.3 kg/m^2^) attending endocrinology and/or nutrition consultations at ULS São João (Porto, Portugal) were assessed regarding quality of life (Diabetes Health Profile) and social desirability (Marlowe–Crowne’s scale). Anthropometric (weight and height), biochemical (A1c) and metabolic control data (time at glycemic target) were collected, as well as information on type and duration of insulin therapy, carbohydrate counting, use of mobile applications for carbohydrate counting and perceived health status. **Results**: Older patients had higher BMI (r = 0.227, *p* = 0.037) and worse quality of life in the ‘barriers to activity’ domain (r = 0.290, *p* = 0.007). In the multivariate analysis, sociodemographic, clinical and quality of life characteristics significantly explained A1c (*p* = 0.046, η^2^_p_ = 0.271) and perceived health status (*p* = 0.012, η^2^_p_ = 0.313). Higher eating self-efficacy significantly explained better perceived health status (*p* = 0.006, η^2^_p_ = 0.105), but no variable independently had a significant effect on glycemic control. **Conclusions**: Glycemic control results from the interaction between various clinical features that must be considered in personalized approaches. Eating self-efficacy significantly explained perceived health status, and may represent a potential target for intervention among people with type 1 diabetes mellitus.

## 1. Introduction

Diabetes mellitus (DM) is a complex, chronic condition in which glucose is not used effectively as a source of energy, resulting in hyperglycemia [1]. It can be categorized as type 1 diabetes mellitus (DM1), type 2 diabetes mellitus (DM2), gestational diabetes mellitus, and other specific types arising from various causes. DM1 (including latent autoimmune diabetes of the adult, LADA) results from the autoimmune destruction of pancreatic beta cells, which generally leads to absolute insulin deficiency and, therefore, the need to obtain it exogenously. It is thought that DM1 represents around 10% of all DM cases and, although there is currently no effective measure to prevent it, it has been hypothesized that, in addition to other factors unrelated to diet, excess weight may represent a risk factor [2,3].

Poor glycemic control in DM is associated with the onset of acute complications, such as diabetic ketoacidosis and severe hypoglycemia. The aim of treatment and management of DM is to prevent the onset of these complications and to reduce the risk of medium-to-long-term complications. Glycemic control can be assessed by measuring glycosylated hemoglobin (A1c), by capillary glucose monitoring or through continuous monitoring systems using the ‘time in range’ metric or through average glucose. A1C values correspond to the proportion of glycosylated (i.e., ‘coated’ with sugar) hemoglobin, and measures the average blood sugar levels over the previous 2 to 3 months. It is a valuable tool for diagnosing and monitoring diabetes and prediabetes, as it indicates the overall blood glucose control. The A1c value is strongly related to the risk of complications from DM [4,5] and current recommendations target a maximum A1c of 7% [6].

Individuals with DM1 require the administration of exogenous insulin. Within intensive insulin therapy, the use of continuous subcutaneous insulin infusion (CSII) seems to be more effective in reducing A1c levels [7,8,9], with patients who had worse control on a regimen involving the multiple administration of insulin analogs benefiting more from the transition to CSII [7,8,9,10]. In addition, the use of CSII is associated with a lower risk of hypoglycemia [7,8,9,10,11] and less glycemic variability [7,8].

In patients with DM1, carbohydrate counting (CHC) is a safe dietary strategy to achieve adequate glycemic control [12], and is associated with improvements in control compared to dietary education strategies alone [13,14,15]. As far as diet therapy is concerned, applications that enable CHC are intended to help patients make a better estimate of the carbohydrate content and, consequently, to administer the correct amount of insulin. The use of applications that enable CHC and the calculation of the insulin bolus to be administered seem to show promising results [15].

DM has various physical, psychological and behavioral implications for quality of life [16]. Good management of this disease requires the active participation of the patient, and it is becoming increasingly important to assess the impact of the disease on the patient’s behavioral profile and quality of life, which seems to be negatively affected by diabetes. Within the insulin therapy available for the treatment of DM, the use of CSII compared to the multiple administration of insulin analogs seems to be associated with better glycemic control [7,17] and a better quality of life [11,18]. The quality of life of those who use the CHC method seems to be better compared to those who do not.

Eating self-efficacy refers to beliefs in the ability to implement the actions necessary to achieve a certain result regarding food and eating, as well as the feeling of control over one’s dietary behaviors and environment [19,20]. This dimension is particularly important because patients with diabetes mellitus are expected to take on a series of behaviors and activities aimed at self-care and good management of this chronic disease on a daily basis. Lower levels of eating self-efficacy are associated with greater resistance to treatment or the adoption of measures [21], which could have implications for glycemic control.

Social desirability corresponds to the tendency of individuals to try to convey a culturally accepted image, in accordance with social norms [22], so this is a potential source of bias when self-report instruments are used.

Despite glycemic control being a fundamental feature of diabetes treatment, the literature is scarce regarding its relationship with eating behavior, namely eating self-efficacy, which may play a major role in determining the ability to comply with dietetic recommendations. On the other hand, a multidimensional perspective of health care provision implies the concomitant consideration of objective measures (such as glycemic control) and subjective measures (namely quality of life) as clinical outcomes. Moreover, the relationships between such features may vary between patients with different sociodemographic and clinical characteristics (namely regarding the use of CHC, CSII and CHC apps), and the knowledge of these differences is of high relevance for the implementation of strategies aimed at improving the efficacy of dietary therapy and glycemic control.

The objectives of this study were (1) to assess glycemic control and quality of life, defined in terms of disease-related psychosocial and behavioral dysfunction (psychological strain, barriers to activity and disinhibition in eating) of adults with DM1; (2) to compare sociodemographic characteristics (sex, age, education), clinical characteristics, glycemic control, quality of life and eating self-efficacy according to the use of CHC, CSII and CHC apps; (3) to study the relationships between sociodemographic and clinical characteristics, glycemic control, quality of life and eating self-efficacy; and (4) to study the effect of sociodemographic, clinical characteristics, quality of life and eating self-efficacy on glycemic control and perceived health status. Based on the current body of knowledge, we expect that younger and more educated participants present better glycemic control and perceived health status. We also hypothesize that these two outcomes are associated with higher eating self-efficacy and better disease-related psychosocial profile.

## 2. Methods

This study used a convenience sample of diabetic patients attending the Nutrition and/or Endocrinology consultation at the Local Health Unit of São João (Porto, Portugal). All the study procedures were approved by the Ethics Committee of the University Hospital Centre of São João/Faculty of Medicine of the University of Porto (Reference 85/2023) and it was carried out under the parameters of the Declaration of Helsinki.

### 2.1. Sample and Procedures

Recruitment took place between August 2023 and January 2024. Inclusion criteria were (i) age between 18 and 65; and (ii) diagnosis of DM1 at least one year previously. Exclusion criteria were the presence of a pathology or clinical condition that implied dietary therapy other than that recommended for the treatment of diabetes and/or that affected the ability to make a free and informed decision or to participate. After providing written informed consent, participants answered a questionnaire collecting sociodemographic (sex, age and education) and clinical data (current glycosylated hemoglobin, time in range, co-morbidities, duration of illness, type of insulin therapy, use of CSII, CHC and mobile applications for CHC), which was verified and complemented with the information available in each patient’s electronic clinical record. Finally, participants were assessed regarding their health-related quality of life, perceived health status, eating self-efficacy and social desirability.

### 2.2. Instruments and Measures

Height was measured using a SECA^®^ 213 portable stadiometer (SECA, Hamburg, Germany) (precision of 0.1 cm). Weight was measured using the InBody body composition analyser model 230 (Seoul, Republic of Korea) (precision of 100 g).

The Portuguese 18-item version of the Diabetes Health Profile [23] was used to assess the impact of living with DM on the emotional and social state. It is an 18-question instrument that aims to outline the psychosocial profile of patients who use insulin, across different dimensions: (1) psychological distress, which comprises 6 items referring to problems such as depressive symptoms, more arguments and hassles at home or feelings of hopelessness and irritability due to diabetes; (2) barriers to activity, which comprises 7 items focusing on issues such as food controlling life or avoiding going out when sugar is low; and (3) disinhibited eating, which comprises 5 items and refers to issues such as finding it easy to stop eating after having started, or wishing there were not so many good things to eat. The items are answered in a Likert-type scale with 4 response options, and the answers in each dimension are summed and transformed into a score between 0 and 100, where 0 indicates no dysfunction and 100 the worst perception of quality of life. The Portuguese version of this instrument presented adequate psychometric properties, namely construct validity, content validity, and reliability (Cronbach’s alpha values for the psychological distress, barriers to activity and disinhibited eating dimensions of, respectively, 0.834, 0.645 and 0.732.

As for the assessment of perceived health status, a visual analog scale from 0 to 100 was used, in which the higher the value, the better the subjective state of health: 0 corresponds to the ‘worst state of health’ and 100 to the ‘best state of health’ [24]. Participants were asked to indicate the number that best described their state of health at the time.

Eating self-efficacy was assessed using the General Eating Self-Efficacy Scale [25], which comprises 5 items with 5 response options ranging from ‘I don’t agree’ to ‘I agree very much’. Each item is graded from 0 to 4 points and the total score (0 to 20 points) is obtained by the sum of the scores of individual items. Higher scores correspond to higher eating self-efficacy. This scale has shown good internal consistency, with Cronbach’s alpha values above 0.85, as well as convergent and discriminant validities, both among higher education students [25] and in a clinical sample [26].

The Portuguese composite version of Marlowe–Crowne’s Social Desirability Scale [27] comprises 12 dichotomous response items (‘Yes’/‘No’), to each of which 1 point is awarded to the response that corresponds to the attitude that reveals social desirability. The total score (0 to 12 points) results from the sum of the scores on the items, and higher scores correspond to greater social desirability. This scale has shown adequate psychometric properties, including internal consistency (Kuder–Richardson index of 0.60), temporal stability (three months test–retest: r = 0.76, *p* < 0.01), discriminant and divergent validity [27].

### 2.3. Statistical Analysis

The statistical analysis was carried out using IBM SPSS version 29.0 for Windows. Descriptive statistics consisted of absolute (*n*) and relative (%) frequencies, means and standard deviations (SDs).

The normality of quantitative variables was assessed using asymmetry and kurtosis. When the variables had a non-normal distribution, a 2-parameter logarithmic transformation was applied along with a linear transformation so that the median was not altered. The transformed variables were used in the inferential analysis, but descriptive statistics are presented for the non-transformed variables.

All inferential statistics were carried out with 95% confidence. Pearson’s correlation coefficient (r) was used to measure the degree of association between pairs of variables, and Student’s *t*-tests and one-way ANOVA were used to compare means of independent samples (post hoc tests were performed with Sidak’s correction). A MANCOVA model was carried out to study the effect of various characteristics on A1c and perceived health status.

## 3. Results

Sample characterization and comparison between sexes:

The final sample consisted of 85 participants, 49.4% of whom were women (*n* = 42). Table 1 shows the characterization of the sample and comparison between sexes. Participants had a mean age of 37 years (SD = 13), mean BMI of 25.8 kg/m^2^ (SD = 3.9), mean education of 14 years (SD = 4) and mean duration of illness of 20.3 years (SD = 12.7).

With regard to analytical values and disease-related data, the average A1C value was 7.7% (SD = 1.2) and the average time in range was 57% (SD = 20). Forty percent of the participants used CSII, with a mean duration of use of 6.6 years (SD = 4.0), 81% performed CHC, with a mean duration of CHC of 7.6 years (SD = 5.6) and 39% used applications, with a mean duration of use of 1.7 years (SD = 1.4).

The average score on the eating self-efficacy scale was 12.9 (SD = 3.6). With regard to the score on the DHP, the quality of life dimension was highest for barriers to activity (mean = 32.0; SD = 17.0), followed by disinhibited eating (mean = 29.7; SD = 16.8) and psychological distress (mean = 18.7; SD = 15.5). The average perceived health status was 72 (SD = 18), and the mean score on the social desirability scale was 8.2 (SD = 2.3).

Regarding the comparison between sexes, significant differences were found only for the ‘Psychological distress’ dimension of the DHP, with women scoring higher than men (mean = 17.3 vs. 15.6; *p* = 0.039).

### Use of CSII, CHC and Apps

Table 2 compares the groups defined by the use of CSII, CHC and apps for CHC in terms of age and education, BMI, clinical parameters, the dimensions of the DHP, eating self-efficacy and social desirability. There were no significant differences between groups regarding health status perception, glycemic control (A1c or time in range), DM duration or eating self-efficacy.

Participants not on CSII and who did not perform CHC were on average 13 to 14 years older than those on CSII with (*p* = 0.039) or without apps (*p* = 0.016) and those using apps but not on CSII (*p* = 0.015). Despite the overall significant difference between groups in terms of education (*p* = 0.017), the post hoc comparisons did not identify significant differences between pairs of groups. The tendency was for participants that were not on CSII and did not perform CHC, or who used apps to perform it, to have lower education than the remaining.

With regard to BMI, participants who were not on CSII and did not perform CHC had on average a BMI 3.9 kg/m^2^ higher than those on CSII and performing CHC with apps (*p* = 0.008).

Concerning the dimensions assessed by the DHP, there were no differences between the groups for disinhibited eating or psychological distress. Patients who were not on CSII and did not perform CHC presented greater dysfunction in terms of barriers to activity than the group with CSII and CHC with apps (mean score = 40.5 vs. 20.4; *p* = 0.012).

Participants without CSII and who performed CHC with apps had the highest mean social desirability score, their mean score being significantly higher than those with CSII and performing CHC with apps (mean difference of 2.3).

Relationships of age and education with anthropometric, clinical and behavioral characteristics:

Table 3 presents the associations between clinical, anthropometric, psychological and behavioral characteristics and age and education. Older individuals had worse perceived health (r = −0.220; *p* = 0.043), higher BMI (r = 0.227; *p* = 0.037), longer duration of illness (r = 0.668; *p* < 0.001), longer duration of CSII use (r = 0.459; *p* = 0.006) and more barriers to activity (r = 0.290; *p* = 0.007). On the other hand, people with a higher level of education had lower A1C values (r = -0.329; *p* = 0.002), fewer barriers to activity (r = −0.224; *p* = 0.040) and lower social desirability (r = −0.310; *p* = 0.004).

Effects of sociodemographic and clinical characteristics, glycemic control, quality of life and eating self-efficacy on glycemic control and health status:

Table 4 shows the effects of sex, age, level of education, BMI, duration of illness, eating self-efficacy, barriers to activity, disinhibited eating, psychological distress and social desirability on the A1C value and perceived health status. Both A1C value and health status were significantly explained by all the variables included in the model, with the effect size being greater for health status (η^2^_p_ = 0.313, *p* = 0.012 vs. η^2^_p_ = 0.271, *p* = 0.046 for A1c).

Only eating self-efficacy significantly explained perceived health status (*p* = 0.006, η^2^_p_ = 0.105), with higher levels of eating self-efficacy being related to a better perception of health status. With regard to A1C, none of the variables independently significantly explained the value.

## 4. Discussion

In this study, we aimed to assess glycemic control, relating it to different dimensions of quality of life and anthropometric and sociodemographic parameters, in order to understand how the quality of life of individuals can be affected by the presence of DM1 and which factors contribute most to glycemic control.

We hypothesized that better glycemic control and perceived health status were related to lower age and higher education. Education level appears to be an important predictor of glycemic control in patients with DM [28,29]. Thus, participants with lower levels of education tend to have worse glycemic control, while higher levels of education appear to be associated with greater skills and knowledge about the disease and its consequences, which may lead patients to make the necessary lifestyle changes, namely improving eating habits, practicing physical activity, adhering to therapy and regularly monitoring blood glucose levels. However, in the bivariate analysis, age only presented a relationship with perceived health status, and education with glycemic control.

Following previous findings [29,30], it was found that older patients had a higher BMI and more barriers to activity. One possible explanation for these findings is that, with advancing age and living with DM (a chronic disease that requires a high level of self-care from the patient) for several years, there may be lower adherence, namely to an adequate CHC or correct administration of insulin, which may result in poorer glycemic control. On the other hand, the fact that the multivariate analysis adjusted the results for possible confounders, namely age, may have influenced these associations.

We did not find any association between longer disease duration and higher A1C values, contrary to what some studies have indicated, where individuals with longer disease duration had higher A1C values compared to those with a more recent diagnosis [28]. Although, in some studies, younger individuals with shorter disease duration, lower BMI and higher education levels had better A1C values [28], in other studies, older age did not appear to be necessarily associated with higher A1C values among people with type 1 diabetes [31].

Eating self-efficacy can be defined as the belief that the individual will be able to perform a certain task required to produce the desired result and the feeling of control over behaviors and the environment [19,32]. No relationship was observed between eating self-efficacy and glycemic control, although some studies suggest that greater eating self-efficacy is associated with better glycemic control [29]. In our study, eating self-efficacy significantly explained perceived health status in the multivariate analysis. The feeling or belief that an individual is not capable of adopting measures to achieve a certain glycemic goal can lead them to have a more negative perception of their health status compared to what their health status actually is in practice.

Patients not on CSII and who did not perform CHC presented greater dysfunction in terms of barriers to activity than the group with CSII and CHC using apps. However, these groups also presented significant age differences, which may explain the lack of significant effect of barriers to activity when age is considered simultaneously. Despite the interest in performing multivariate analysis, namely to clarify possible causal relationships, we highlight that the bivariate relationships may also be useful for guiding future research. Considering the scarce research on how eating self-efficacy and disease-related psychosocial profile may interact in terms of glycemic control and perceived health status in people with type 1 diabetes with different clinical features, future research should try to address the study of such relationships, namely with larger samples, which will allow more accurate comparisons.

This study has some limitations that should be considered when interpreting the results, as well as for future studies, such as the fact that the medication used by the patients was not collected, which may have obscured some altered analytical values. The fact that a convenience sample was used should be seen as a limitation, but this aspect is partially offset by the fact that the sample was collected in a public hospital, where people with diverse sociodemographic characteristics are treated and where access is provided to a wider group of the population [33]. The lack of time in range data for all patients prevented a more complete analysis with regard to glycemic control and its relationships with other variables. The main strengths of our work are the sample size, an identical proportion of men and women in our sample, as well as a combined assessment of several clinical, sociodemographic, behavioral and quality of life features.

## 5. Conclusions

This work contributes to a broader understanding of the factors that may impact the quality of life of people with DM1, as well as to understanding the determinants of glycemic control in this population. Our results indicate that glycemic control, as defined by A1C values, is the result of the interaction between various clinical features that patients with DM1 should consider, aiming for optimal metabolic control and prevention or delay of the onset of complications. Furthermore, eating self-efficacy was the only variable that significantly explained perceived health status, which may represent a focus for intervention among people with DM1.

## Figures and Tables

**Table 1:** Sample characterization and comparison between sexes.

	Total Sample (*n* = 85)	Women (*n* = 42)	Men (*n* = 43)	*p*
	Mean (SD)	Mean (SD)	Mean (SD)	
Age (years)	37 (13)	35 (12)	38 (13)	0.356
Education (years)	14 (4)	14 (3)	14 (4)	0.914
BMI (kg/m^2^)	25.8 (3.9)	25.8 (2.7)	24.8 (3.7)	0.169
A1C (%)	7.7 (1.2)	7.8 (1.1)	7.6 (1.2)	0.510
Time in range (%) [*n* = 64]	57.0 (20.1)	54.9 (20.1)	59.1 (20.2)	0.402
Diabetes duration (years)	20.3 (12.7)	21.5 (12.1)	19.1 (13.3)	0.385
CSII duration (years) [*n* = 34]	6.6 (4.0)	7.2 (4.6)	5.8 (2.9)	0.300
CHC duration (years) [*n* = 69]	7.7 (5.6)	8.3 (5.3)	7.0 (6.0)	0.334
App duration (years) [*n* = 33]	1.7 (1.4)	1.2 (1.1)	2.0 (1.5)	0.119
Health status	71.6 (17.6)	74.4 (3.8)	75.3 (3.4)	0.254
Barriers to activity	32.0 (17.0)	32.7 (17.6)	31.5 (16.6)	0.747
Disinhibited eating	29.7 (16.8)	32.5 (16.5)	27.0 (16.9)	0.128
Psychological distress	18.7 (15.5)	17.3 (3.9)	15.6 (3.7)	0.039
Eating self-efficacy	12.9 (3.6)	12.7 (3.5)	13.0 (3.7)	0.692
Social desirability	8.2 (2.3)	8.3 (2.3)	8.1 (2.3)	0.598

A1C: Glycosylated hemoglobin. BMI: Body mass index. CHC: Carbohydrate counting. CSII: Continuous subcutaneous insulin infusion. Health status, barriers to activity, disinhibited eating and psychological distress: score between 0 and 100. Eating self-efficacy: score between 0 and 20. Social desirability: score between 0 and 12.

**Table 2:** Comparison of sociodemographic, clinical, glycemic control, anthropometric, quality of life and eating self-efficacy according to the use of CSII, CHC and apps.

	No CSII, No CHC	No CSII, CHC, No Apps	No CSII, CHC with Apps	CSII, CHC, No Apps	CSII, CHC with Apps	*p*
	(*n* = 16)	(*n* = 16)	(*n* = 19)	(*n* = 20)	(*n* = 14)	
	Mean (SD)	Mean (SD)	Mean (SD)	Mean (SD)	Mean (SD)	
Age (years)	47 (14) ^a^	33 (11) ^b^	37 (11) ^ab^	34 (11) ^b^	34 (13) ^b^	0.007
Education (years)	12 (5) ^a^	14 (4) ^a^	12 (3) ^a^	15 (3) ^a^	15 (3) ^a^	0.017
BMI (kg/m^2^)	27.2 (3.5) ^a^	25.0 (1.9) ^ab^	24.5 (4.2) ^ab^	26.2 (2.9) ^ab^	23.3 (1.9) ^b^	0.007
Health status	74.1 (3.5) ^a^	75.7 (4.3) ^a^	74.7 (2.7) ^a^	75.0 (4.4) ^a^	74.7 (3.3) ^a^	0.842
A1C (%)	8.1 (1.4) ^a^	7.3 (0.8) ^a^	7.9 (1.3) ^a^	7.9 (1.3) ^a^	7.3 (0.6) ^a^	0.123
Time in range (%) (*n* = 64)	45.8 (22.2) ^a^	58.5 (15.2) ^a^	54.4 (23.1) ^a^	57.8 (22.5) ^a^	64.8 (14.3) ^a^	0.328
DM duration (years)	25.8 (16.9) ^a^	14.3 (8.4) ^a^	17.8 (11.9) ^a^	22.6 (13.0) ^a^	20.7 (9.4) ^a^	0.089
Disinhibited eating	29.2 (22.0) ^a^	26.3 (15.1) ^a^	35.1 (16.0) ^a^	32.7 (15.7) ^a^	22.9 (13.0) ^a^	0.234
Psychological distress	16.5 (3.7) ^a^	15.9 (4.2) ^a^	16.5 (3.9) ^a^	16.2 (4.4) ^a^	17.3 (3.4) ^a^	0.903
Barriers to activity	40.5 (17.2) ^a^	33.3 (15.2) ^ab^	31.6 (16.1) ^ab^	32.9 (15.7) ^ab^	20.4 (17.8) ^b^	0.028
Eating self-efficacy	12.6 (4.0) ^a^	13.9 (3.2) ^a^	12.2 (3.4) ^a^	12.2 (3.5) ^a^	14.1 (3.6) ^a^	0.358
Social desirability	8.3 (1.8) ^ab^	8.8 (2.0) ^ab^	9.1 (2.6) ^a^	7.8 (2.4) ^ab^	6.8 (2.0) ^b^	0.038

The presence of the same letter in superscript indicates the absence of significant differences in the post hoc tests. A1C: Glycosylated hemoglobin. BMI: Body mass index. CHC: Carbohydrate counting. CSII: Continuous subcutaneous insulin infusion. Health status, barriers to activity, disinhibited eating and psychological distress: score between 0 and 100. Eating self-efficacy: score between 0 and 20. Social desirability: score between 0 and 12.

**Table 3:** Relationship between sociodemographic (age and education) and clinical characteristics, glycemic control, quality of life, eating self-efficacy and social desirability.

	Age r (*p*)	Education r (*p*)
BMI (kg/m^2^)	0.227 (0.037)	−0.083 (0.105)
A1C (%)	0.034 (0.755)	−0.329 (0.002)
Time in range (%) [*n* = 64]	0.081 (0.526)	0.224 (0.075)
Health status	−0.220 (0.043)	−0.014 (0.900)
DM duration (years)	0.668 (<0.001)	−0.030 (0.785)
Pump duration (years) [*n* = 34]	0.459 (0.006)	0.027 (0.879)
CHC duration (years) [*n* = 69]	0.013 (0.917)	0.147 (0.227)
App duration (years) [*n* = 33]	−0.112 (0.535)	0.321 (0.069)
Disinhibited eating	−0.084 (0.446)	−0.136 (0.214)
Psychological distress	−0.034 (0.756)	−0.122 (0.267)
Barriers to activity	0.290 (0.007)	−0.224 (0.040)
Eating self-efficacy	−0.152 (0.165)	0.199 (0.068)
Social desirability	0.085 (0.437)	−0.310 (0.004)

A1C: Glycosylated hemoglobin. BMI: Body mass index. CHC: Carbohydrate counting. Health status, barriers to activity, disinhibited eating and psychological distress: score between 0 and 100. Eating self-efficacy: score between 0 and 20. Social desirability: score between 0 and 12.

**Table 4:** Multivariate effects of sociodemographic and clinical characteristics on A1C and perceived health status (*n* = 85).

	A1C (%)			Perceived Health Status		
	β	*p*	η^2^_p_	β	*p*	η^2^_p_
Corrected model		0.046	0.271		0.012	0.313
Female sex	0.008	0.977	0.000	−0.641	0.435	0.009
Age (years)	−0.023	0.109	0.036	−0.020	0.643	0.003
Education (years)	−0.063	0.109	0.036	−0.131	0.276	0.017
Group		0.455	0.050		0.837	0.020
CSII, CHC with apps	(Ref.)			(Ref.)		
CSII, CHC without apps	0.308	0.478		0.941	0.482	
No CSII, CHC with apps	0.201	0.650		−0.371	0.786	
No CSII, CHC without apps	−0.309	0.500		0.101	0.943	
No CSII, no CHC	0.373	0.441		−0.323	0.829	
BMI (kg/m^2^)	−0.004	0.925	0.000	−0.041	0.758	0.001
DM duration (years)	0.018	0.198	0.024	−0.039	0.352	0.012
Barriers to activity	0.017	0.072	0.045	−0.005	0.861	0.000
Disinhibited eating	−0.001	0.895	0.005	−0.000	0.998	0.000
Psychological distress	0.022	0.571	0.005	−0.214	0.083	0.042
Eating self-efficacy	−0.025	0.547	0.005	0.358	0.006	0.105
Social desirability	0.065	0.312	0.015	0.220	0.267	0.018

A1C: Glycosylated hemoglobin. BMI: Body mass index. CHC: Carbohydrate counting. CSII: Continuous subcutaneous insulin infusion. Health status, barriers to activity, disinhibited eating and psychological distress: score between 0 and 100. Eating self-efficacy: score between 0 and 20. Social desirability: score between 0 and 12.

## Data Availability

Data supporting the conclusions of this article will be made available by the authors on reasonable request.
